# Using Functional Near-Infrared Spectroscopy to Elucidate Neurophysiological Mechanism of Action of Equine-Assisted Services: Proof-of-Concept Study

**DOI:** 10.3390/ijerph22081294

**Published:** 2025-08-19

**Authors:** Beth A. Lanning, Cory M. Smith, Cierra Ugale, Elena Nazarenko, William R. Marchand

**Affiliations:** 1 Department of Public Health, Baylor University, Waco, TX 76655, USA; 2 Department of Health, Human Performance and Recreation, Baylor University, Waco, TX 76655, USA; cory_m_smith@baylor.edu (C.M.S.); cierra_ugale1@baylor.edu (C.U.); 3 Whole Health Service, VA Salt Lake City Health Care System, 500 Foothill Drive, Salt Lake City, UT 84108, USA; elena.nazarenko@va.gov (E.N.); william.marchand@va.gov (W.R.M.); 4 Department of Psychiatry, University of Utah, 501 Chipeta Way, Salt Lake City, UT 84108, USA; 5 Department of Animal, Dairy and Veterinary Sciences, Utah State University, 0500 Old Main Hill, Logan, UT 84322, USA

**Keywords:** equine-assisted services, veterans, fNIRS, neurophysiology, psychological flexibility, PTSD

## Abstract

Equine-assisted services (EAS) are used for civilian and military trauma survivors to reduce depression and posttraumatic stress symptoms. While early scientific evidence supports the benefits of EAS, the neurophysiological mechanisms underlying these benefits are unknown. The specific aims of this exploratory study were to determine (1) whether functional near-infrared spectroscopy (fNIRS) neuroimaging can be used to explore neural responses of EAS veteran participants and (2) the correlation between neural responses and psychological outcomes of the participants interacting with equines. Fifteen veterans participated in a 2-day EAS program consisting of four randomized activities. An fNIRS sensor cap was used to measure the oxygenated (O_2_Hb), deoxygenated (hHb), and total hemoglobin (tHb) of the participants during each activity. The results indicated no significant differences for O_2_Hb and tHb across the visits or activities, however, a significant difference in hHb was observed. There was an increase in hHb during the activities that included an equine, which indicated a greater cognitive load and attention. Further, data from pre-/post-psychometric assessments showed a significant improvement in participants’ trait anxiety, psychological flexibility, and positive and negative affect after interacting with the horse. Preliminary data revealed a potential association between the cognitive attention and psychological health of participants during an EAS session.

## 1. Introduction

Post-traumatic stress disorder (PTSD) is a psychiatric condition that affects approximately 3.5% of U.S. adults every year. While the reported rates of PTSD vary across populations and studies, conservative estimates show 6% of the general population may experience PTSD in their lifetime and 7% of veterans may experience PTSD in their lifetime, with the prevalence being higher among younger veterans (9–15%) than those over age 65 (4%) [1]. Individuals diagnosed with PTSD often experience reoccurring intrusive thoughts, hypervigilance, flashbacks, nightmares, emotional numbing, withdrawal, difficulty concentrating, negative affect, and difficulty sleeping [2]. Co-occurring conditions such as mood and anxiety disorders, self-harm, and impulsive behavior are also common among individuals diagnosed with PTSD [3].

The treatment options for veterans suffering from PTSD and co-occurring conditions vary from recommended trauma-focused psychotherapies (cognitive processing therapy, eye movement desensitization and reprocessing, prolonged exposure therapy) [4] to complementary and alternative options (e.g., recreational therapy, equine-assisted services, music therapy), all of which are designed to reduce a patient’s PTSD symptoms and improve their well-being [5,6]. While trauma-focused therapies have been considered the gold standard for individuals suffering from PTSD, the effectiveness rate of these therapies for military veterans is less than 50% [7]. Further, veterans suffering from PTSD often struggle with completing the therapy sessions and adhering to therapy protocols, which reduces the potential for improvement and potentially exacerbates negative mental health outcomes [8]. In response, alternative and complementary interventions, such as equine-assisted services, have increased in popularity and are being promoted as providing therapeutic benefits for the participants.

Equine-assisted services (EAS) is a categorical term for interventions that include a human–horse interaction designed to provide therapeutic benefits for the participants. EAS include equine-assisted learning, therapeutic riding, and psychotherapy that incorporates horses [9]. Typically considered complementary to more traditional therapies, EAS are increasingly being used as interventions for both civilians and military populations with PTSD. A growing body of evidence suggests that EAS may be helpful in reducing anxiety, decreasing depression and PTSD symptoms, and increasing the quality of life for veterans and military service members [10]. While promising, the mechanism of change is still unclear. Proponents and participants cite the interactive relationship between the person and the horse as a possible theory of change. It is believed that the horse, as a prey and herd animal, provides a type of mirror effect for the participant’s emotions and body language [11]. As prey animals, horses have evolved to be extremely sensitive to their environment to avoid predators. These instincts facilitate awareness of both verbal and non-verbal cues from the participant, which, in turn, provides the participant immediate feedback on their emotional state and communication skills [12,13]. Other working theories are that the horse–human interaction can provide comfort and/or emotional safety for the participant through the development of a nonjudgemental relationship [14,15]. See Marchand et al. [16] for a more complete discussion of working theories. Although many of the theories show potential, they have not yet been thoroughly validated through rigorous research, and much of the reported therapeutic change relies primarily on self-reported psychosocial outcomes [17]. Future research examining physiological and neurological changes related to the psychosocial outcomes is needed to fully understand the possible benefits of the human–equine interaction and to elucidate the possible mechanism(s) of change [17,18].

While some research using more objective assessments such as heart rate variability and cortisol levels among EAS participants is emerging [19,20], we are aware of only one EAS study that included neuroimaging outcomes [21]. In that study, the researchers used functional magnetic resonance imaging (fMRI) to examine potential neurological changes among a subset of veterans who participated in a psychotherapy program that integrated equines. Their findings indicated that a reduction in PTSD symptoms was correlated with changes in the functional connectivity of the brain. Human neuroimaging techniques, including high-resolution magnetic resonance imaging (MRI) and functional magnetic resonance imaging (fMRI), have been used to assess structural neurological changes due to PTSD [22] and brain function before and after receiving an eight-week EAS intervention for PTSD [21]. While effective and considered the gold standard in neuroimaging, the cost and functional limitation associated with these methods make them impractical for field-based settings such as horse arenas where EAS are typically provided. Further, these modalities do not provide information regarding real-time changes in brain activation during EAS sessions.

Functional near-infrared spectroscopy (fNIRS) has emerged as a non-invasive, portable, safe, and cost-effective means to monitor brain activity in various settings. Balters et al. [23] used fNIRS to assess emotion-evoked cortical activation patterns in youth with PTS symptoms (PTSS), finding a strong correlation between cortical responses and PTSS scores. Further, Gramlich et al. [24], using fNIRS, reported that veterans with PTSD exhibited increased activation of the right superior/middle frontal gyrus (sections of the brain that play a key role in self-awareness and memory retrieval) when exposed to a threat memory. More recently, Kalanadhabhatta et al. [25] used fNIRS to investigate whether performance on cognitive tasks, physiological changes, and prefrontal cortex, together, could be used to identify individuals with PTSD. Their findings supported the use of fNIRS in community-based settings. Further, fNIRS is comparable to fMRI in terms of demonstrating brain activation but is much more suited to EAS research given its portability and its ability to capture brain activation during EAS participation.

The first aim of this proof-of-concept study was to determine whether fNIRS methodology could be used safely in an EAS setting. Given that horses are prey animals and may become frightened by unfamiliar objects, it is possible that the fNIRS equipment might create a dangerous situation for equines and humans. Additionally, it is possible that the equipment could trigger anxiety and/or exacerbation of PTSD among participants. The second aim was to determine if the fNIRS technology has the potential to elucidate neurophysiological mechanisms underlying the benefits of EAS for veterans with PTSD. Specifically, we aimed to determine whether fNIRS methodology can be used to explore real-time neural responses to EAS participation. If so, this methodology should not only help to disambiguate the general neural mechanisms underlying EAS benefits in this population but also help to parse neural responses to specific EAS activities. Such findings could ultimately inform the design of more effective interventions by indicating which activities result in desired changes in activation. The final aim was to determine if fNIRS results would correlate with psychological instrument outcomes and thus provide more robust information regarding the neural mechanisms underlying the benefits of EAS.

## 2. Methods

### 2.1. Recruitment

This study was conducted as a collaboration between a large urban Veterans Health Administration (VHA) medical center, two universities, and an equine facility. Recruitment and subject enrollment occurred at the VHA medical center. Data collection occurred at the equine facility while the data analyses and writeup occurred at the two universities. The data collection occurred over a one-week period in May of 2024. Demographic and diagnostic information was extracted from the patients’ electronic heath records (EHRs), additional demographic information was collected using a locally developed survey, and the data from pre- and post-intervention psychological measures and fNIRS were collected during the intervention.

### 2.2. Participants and Procedures

Potential subjects were informed of the project by VA medical center staff and those that expressed interest in participating were referred to study staff for an evaluation. Inclusion criteria were male, veterans, currently enrolled for services at the medical center, diagnosis of PTSD, and age range of 21 to 55 years old. In this pilot study, only males were enrolled to avoid sex-related confounding factors in the outcome variables [26,27,28,29]. Exclusion criteria were a diagnosis of cognitive impairment or psychotic spectrum illness, active substance use disorder, current use of beta-blockers or other heart rate limiting medications, and/or a history of traumatic brain injury within the past 6 months. Study staff reviewed the VHA EHR of potential participants. Those that met the criteria were contacted and offered the opportunity to participate in this study. Those that agreed were consented and enrolled.

Each participant was scheduled to complete two 40-minute sessions on separate days during the week of data collection. Session times (between 10:00 a.m. and 4:30 p.m.) were determined by participant availability, and we did not standardize the time of day or directly evaluate the potential impact of timing on neural activation. The intervention session consisted of four experimental conditions, which were identical across the two sessions (days). These were the following: (A) equine grooming; (B) equine groundwork; (C) mindfulness meditation; and (D) processing/discussion. These components were chosen because they are common across EAS programs [16], and a goal of this study was to determine if fNIRS methodology could be utilized to assess human brain activation of EAS participants while they engage in these activities in various interventions. Further, these components are specifically used in one manualized psychotherapy incorporating horses (PIH) intervention [30] and a secondary aim was to begin to understand neural processes associated with this EAS model. Each component was facilitated by two staff members, a mental health professional (MH), and an equine specialist (ES). To ensure consistency, the same staff members facilitated all sessions for all participants. Details of the intervention are outlined in Table 1. Scripts were used by the facilitators, and an intervention observation fidelity tool was completed by an observing staff member to ensure fidelity and consistency. The order of activities was randomized.

Four equines were utilized in this study and were randomized to the conditions. The four horses utilized for this study were all quarter horse geldings ranging in age from 13 to 20 years old. All had been trained to be handled and ridden western style, including participating in the ground-based activities used in this study. Further, all were accustomed to doing the study activities calmly with humans they had not previously met in the context of horsemanship lessons and therapeutic activities. This study was conducted in the area where all of the horses normally live. The intervention facilitators were well-known to the horses. Two of the study staff and the participants were previously unknown to the horses.

The mental health professional (MH) for this study was a board-certified VHA psychiatrist who was a trained and experienced mindfulness facilitator. To ensure safety of participants, staff, and equines, a Professional Association of Therapeutic Horsemanship, International [31]-certified Equine Specialist in Mental Health and Learning was present at all sessions and relevant equine industry standards were followed. The MH was available during and after each session to respond to any psychological distress exhibited by the human participants.

Outcome measures for this study were safety assessments, physiological results provided by fNIRS monitoring throughout each intervention session, and psychological instruments administered immediately pre- and post-session. Human safety was assessed by having participants complete pre- and post-session psychological instruments (described below) to determine if session participation was associated with either increased anxiety or negative affect. Further, at least three staff members observed each session. Any adverse or potentially adverse effects to participants, staff, or equines were noted. The observation process specifically focused on determining if any signs of distress were displayed by human participants or equines. Signs of human distress included statements of distress, facial expressions indicating discomfort, obvious tremor, or prematurely discontinuing an activity. Signs of equine distress included spooking (moving rapidly away from a feared object), excessive head raising, visible sclera, or tense facial muscles. Lastly, human participants had the opportunity to bring up any uncomfortable experiences during the discussion portion of the activities.

### 2.3. Measures

#### 2.3.1. Neurological Measurements

Prefrontal cortex activation was measured using two continuous-wave fNIRS sensors (PortaLite MKII, Artinis Medical Systems B.V., Elst, The Netherlands) bilaterally attached to the forehead approximately 2 cm above the eyebrows. The sensors were secured to the skin using double-sided adhesive tape and then wrapped with a black elastic pre-wrap bandage to further secure the sensors to the forehead and protect the sensors from ambient light contamination. The placement of the sensors corresponded to the two frontopolar (Fp1 and Fp2) regions of the prefrontal cortex, based on the international 10–20 system. Each sensor consisted of three light-emitting diodes, which emit light at two wavelengths (760 nm and 850 nm), and two detectors placed at inter-optode distances of 29, 35, and 41 mm. Each sensor outputs 3 channels for a total of 6 channels. Data were imported into NI Labview and trimmed for the periods of interest during each session in this study. Preprocessing consisted of channel rejection, motion correction, and temporal filtering (0.01, 0.40 Hz). After pre-processing, the relative concentration changes in oxygenated (O_2_Hb), deoxygenated (hHb), and total hemoglobin (tHb) were calculated for each of the 6 channels. Each hemisphere’s 3-channel concentration values were then averaged together to produce a regional concentration value for the left and right hemisphere. All signals were collected at a sampling rate of 25 Hz in Artinis Oxysoft 4 fNIRS acquisition software. The fNIRS methodology was utilized in this study as it is robust to movement and will allow for the quantification of the metabolic demands occurring within the brain during stationary and dynamic movement tasks [32,33,34].

#### 2.3.2. Psychological Assessments

Three instruments were used to measure immediate pre- to post-session psychological changes. These were the Acceptance and Action Questionnaire II [35], the State-Trait Anxiety Inventory -6 [36], and the Positive and Negative Affect Scale [37].

The Acceptance and Action Questionnaire II (AAQ-II) measures psychological flexibility [35]. This is a 10-item, 7-point Likert scale and lower scores indicate greater psychological flexibility and range from 10 to 70. The 3- and 12-month test–retest reliability is 0.81 and 0.79, respectively [35]. The State-Trait Anxiety Inventory (STAI: Y—6 item) measures state anxiety [36]. The STAI: Y—6 item is a 6-item instrument that measures state anxiety. Respondents answer questions, for example, “I feel calm”, with options of “not at all,” “somewhat,” “moderately,” and “very much.” Some items are reverse scored such that higher scores equal higher levels of state anxiety. The Positive and Negative Affect Scale (PANAS) is a 20-item instrument that measures both positive and negative affect [37]. Respondents answer the question “Indicate the extent that you have felt this way” for items such as “interested” and “excited” on a five-point scale ranging from “very slightly or not at all” to “extremely.” Ten of the items measure positive and the other ten evaluate negative affect and the scoring results in both positive and negative affect scores. For positive affect, higher scores equal increased positive affect. For negative affect, lower scores indicate decreased negative affect. The participants completed the self-assessments before (Time 1) and after (Time 2) each day’s (visit) activities (Visit 1(a) and Visit 2(b)).

### 2.4. Statistical Analysis

#### 2.4.1. Psychological Assessments Analysis

Paired-sample *t*-tests were conducted to evaluate the impact of the intervention on STAI and AAQ scores. PANAS-SF negative affect and positive affect scores were analyzed separately. The positive affect scores followed a normal distribution pattern and were analyzed using a paired-sample *t*-test. However, the negative affect scores violated the Shapiro–Wilk test of normality and were analyzed using the Wilcoxon signed-rank non-parametric test. Repeated measures ANOVAs were conducted to determine if a change in scores for each assessment was affected by the day or visit. A priori power analysis revealed a sample size *n* = 15 was adequate to determine a medium (0.50) effect size with power of 0.80.

#### 2.4.2. fNIRS Data Analysis

Two generalized linear mixed-effects regression models were utilized with fixed effects for time, psychological and baseline fNIRS_amp_, and fNIRS_fc_ (pre-intervention for day one) factors and random-effects intercepts for participants were used to examine fNIRS_amp_ and fNIRS_fc_ factors. The time was coded as a categorical variable (post-intervention for day one and post-intervention for day two). Normal or alternative outcomes models (Bernoulli, Poisson, negative binomial, gamma) were used as appropriate for each response variable. An unstructured covariate model accounted for correlation among outcome measurements among repeated visits. Effect sizes, 95% confidence intervals, and *p*-values are reported. Statistical significance was evaluated at a 0.5 level using two-tailed tests. Three separate 4 (condition: Meditation, Leading, Groom, and Processing) x 2 (Region: Left and Right Hemisphere) x Time (visit: Time 1 and Time 2) repeated measures ANOVAs were performed on oxygenated hemoglobin (O_2_Hb), deoxygenated hemoglobin (hHb), and total hemoglobin (tHb) to determine cerebral blood flow change. Follow-up two- and one-way ANOVAs, as well as post-hoc paired sampled *t*-tests with Tukey-LSD, were performed when appropriate. If sphericity was violated, the Greenhouse–Geisser correction was used. An alpha of *p* ≤ 0.05 was considered statistically significant for all statistical analyses (IBM SPSS Version 25.0, Armonk, NY, USA).

An a priori power analysis from the fNIRS neuroimaging techniques indicated that *n* = 14 is required to reach a power of 0.80 based on the fNIRS amplitude of the deoxygenated hemoglobin changes during cognitive motor skills in a healthy, male population (effect size: 0.76, alpha: 0.05, correlation among measures of 0.50).

## 3. Results

### 3.1. Subjects

The subjects were 15 male veterans with a diagnosis of PTSD. All had a military-related disability, and most were white and had experienced at least one combat deployment. Additional demographic and diagnostic details are outlined in Table 2.

### 3.2. Psychometric Instrument Results

The results of the three psychometric assessments, the STAI-20, PANAS-SF, and AAQ-II, revealed improvement across all measures. There was a statistically significant decrease in STAI scores that had a large effect size on Visit 1 (M = −5.467, SD = 2.92, d= −1.86) and Visit 2 (M = −4.571, SD = 1.91, d = 2.39. The AAQ scores decreased significantly as well, with large effect sizes on both visits: Visit 1 (M = −3.667, SD = 4.419, d = −0.830) and Visit 2 (M = −4.071, SD = 4.565, d = −0.882)

Analysis using paired *t*-tests showed a statistically significant increase in the positive affect scores (PANAS-SF) on Visit 1 (M = 5.143, SD = 3.959, d = 1.299) and Visit 2 (M = 6.769, SD = 6.610, d = 1.024), with large effect sizes. Data from the PANAS-SF negative affect scores did not follow a normal distribution due primarily to several outlier scores. Therefore, a Wilcoxon signed-rank test was conducted. The results indicated a significant decrease in negative affect scores and large effect sizes during both visits (Z = −3.20, *p* = 0.001, r = 0.82; Z = −3.006, *p* = 0.002, r = 0.81, respectively), where the effect size was calculated using r=Z/√n. See Table 3 for the full details.

Repeated measures ANOVAs were conducted to determine if the intervention day/visit (Visit 1, Visit 2) affected the outcomes. There was no significant interaction between the visit and the pre/post scores of all variables, meaning that there was no carryover effect from Visit 1 to Visit 2. The STAI scores had a Wilk’s lambda = 0.97, F (1, 27) = 0.937, *p* = 0.34; the AAQ scores had a Wilk’s lambda = 0.98, F (1, 27) = 0.059, *p* = 0.81; the PANAS positive scores had a Wilk’s lambda = 0.98, F (1, 25) = 0.62, *p* = 0.44; the PANAS negative scores had a Wilk’s lambda = 0.99, F (1, 27) = 0.02, *p* = 0.89. While caution should be used in interpreting these findings due to the small sample size, examination of the profile plots for each assessment confirmed the lack of interaction between the pre- and post-visit scores.

### 3.3. Safety Results

The findings from the pre- and post-session psychological instruments, reported above, suggested that particpation was not associated with increased anxiety or negative affect. Further, there were no observations by staff during the sessions that suggested human discomfort and none of the subjects mentioned psychological or physical discomfort during the discussion portions of the sessions. Lastly, there were no observations of equine distress or incidents of equine behavior that could have been dangerous to praticpants.

### 3.4. fNIRS Results

**Figure 1:**
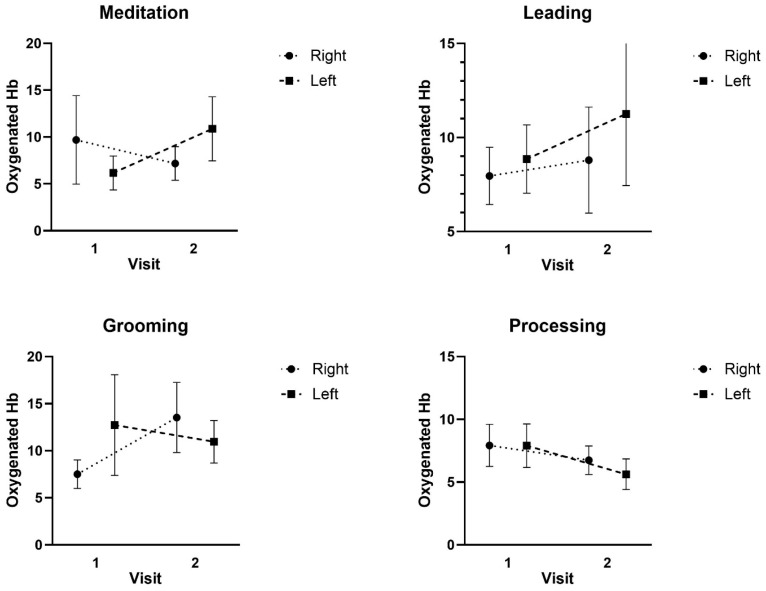
Mean ± SD of the Oxygenated hemoglobin (Hb) responses at Visit 1 and Visit 2 during the Meditation, Leading, Grooming, and Processing tasks.

For O_2_Hb, there was no significant three-way interaction for Condition x Region x Time (F: 0.783; *p* = 0.417; ES = 0.066) or two-way interaction for Time x Region (F: 0.774; *p* = 0.398; ES = 0.067), Condition x Region (F: 0.493; *p* = 0.544; ES = 0.043), or Condition x Time (F: 1.898; *p* = 0.186; ES = 0.147). In addition, there were no significant main effects for O_2_Hb Region (F: 1.112; *p* = 0.314; ES = 0.092), Time (F: 0.230; *p* = 0.641; ES = 0.020), or Condition (F: 0.319; *p* = 0.688; ES = 0.028). This means that there was no detectible difference in oxyhemoglobin across activities, between brain regions, or across time (see Figure 1).

**Figure 2:**
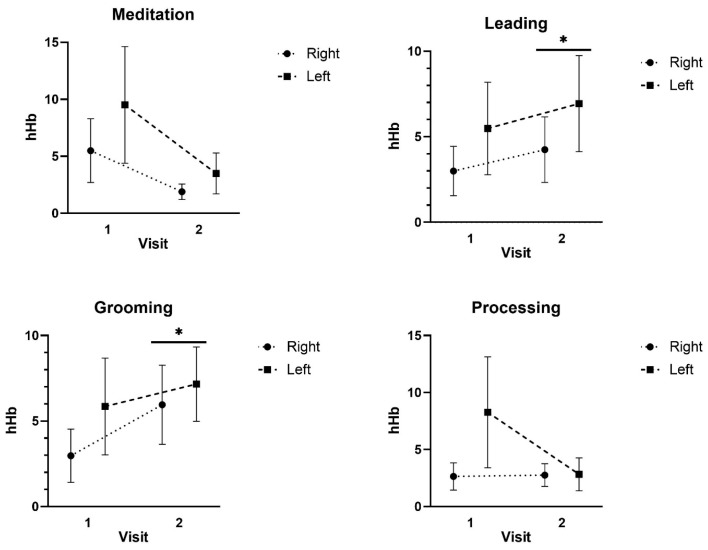
Mean ± SD of the Deoxygenated hemoglobin (hHb) responses at Visit 1 and Visit 2 during the Meditation, Leading, Grooming, and Processing tasks. * Indicates Visit 2 was significantly greater than Visit 1.

For hHb, there was no significant three-way interaction for Condition x Region x Time (F: 0.470; *p* = 0.577; ES = 0.041) or two-way interaction for Time x Region (F: 0.738; *p* = 0.409; ES = 0.063) or Condition x Region (F: 0.047; *p* = 0.940; ES = 0.004). There was, however, a significant two-way interaction for Condition x Time (F: 2.941; *p* = 0.037; ES = 0.288). Follow-up analyses for hHb indicated that there were no detectable differences for the Meditation (*p* = 0.149–0.851) or Processing conditions (*p* = 0.216–0.644). Leading and Grooming exhibited the same pattern of responses, exhibiting significantly greater effects on the hHb compared to Meditation (*p* = 0.019–0.047) and Processing (*p* = 0.041–0.045). In addition, the Time 2 hHb was significantly greater than that of the Time 1 hHb for the Leading (*p* = 0.024–0.045) and Grooming activities (*p* = 0.035–0.041). Differences in deoxyhemoglobin were detected across activities, with the leading and grooming activities generating higher brain activity, and between Visit 1 and Visit 2 (see Figure 2).

**Figure 3:**
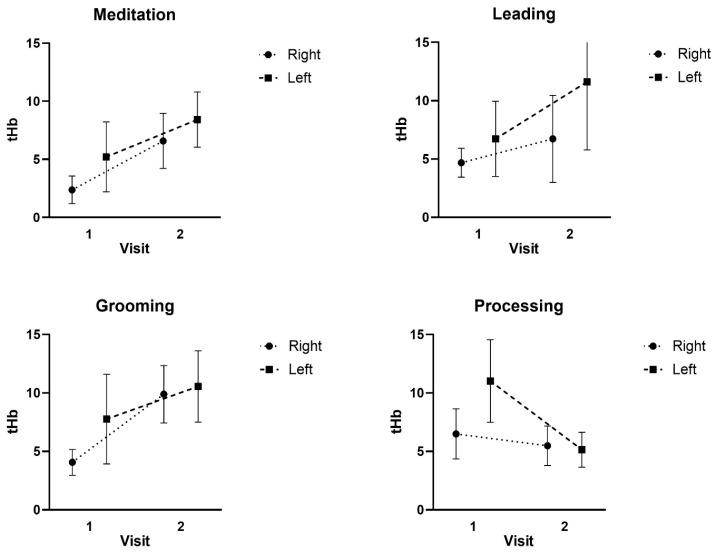
Mean ± SD of the Total hemoglobin (tHb) responses at Visit 1 and Visit 2 during the Meditation, Leading, Grooming, and Processing tasks.

For the tHb, there was no significant three-way interaction for Condition x Region x Time (F: 1.105; *p* = 0.342; ES = 0.091) or two-way interaction for Time x Region (F: 0.006; *p* = 0.940; ES = 0.001), Condition x Region (F: 0.496; *p* = 0.608; ES = 0.043), or Condition x Time (F: 1.083; *p* = 0.358; ES = 0.090). In addition, there were no significant main effects for tHb Region (F: 0.583; *p* = 0.462; ES = 0.050), Time (F: 0.189; *p* = 0.672; ES = 0.017), or Condition (F: 0.056; *p* = 0.915; ES = 0.005) (see Figure 3).

## 4. Discussion

The primary aim of this exploratory study was to determine if functional near-infrared spectroscopy (fNIRS) could be safely utilized to evaluate human brain function during participation in EAS activities. A secondary goal was to assess whether changes in human brain activation demonstrated by fNIRS could be correlated with measures of anxiety, affect, and/or psychological flexibility. A tertiary aim was to begin to elucidate the neural mechanisms associated with the benefits of EAS participation.

The first key finding of this study was that fNIRS methodology proved to be a feasible and reliable method of capturing neural activity during human participation in four non-mounted activities that are common to EAS interventions (equine grooming, equine leading, mindfulness meditation, and discussion). While other studies used fMRI [21,38], our study is the first to utilize fNIRS methodology to evaluate neural activity during EAS participation. MRI-based methodologies, such as functional MRI (fMRI), can provide important information about brain activation and connectivity changes associated with EAS participation, but they cannot establish which EAS activities contribute to specific changes in brain function. Further, they can only demonstrate associations, not cause and effect. Our findings suggest that fNIRS methodology may play an important role in expanding our understanding of the neural mechanisms underlying benefits associated with EAS. Further, these results demonstrate that brain activation changes occur during EAS and that distinct patterns of activation are associated with specific tasks. Research using fNIRS has the potential to disambiguate which specific activities are associated with benefits and thus guide the development of more effective interventions.

In addition to demonstrating that fNIRS can provide important information about brain activation during EAS participation, our findings also demonstrate that this methodology can be utilized safely. While previous studies have demonstrated that fNIRS can be utilized during human physical activities [39,40], EAS presents unique challenges. EAS involve equines, which are prey animals [41]. To avoid being eaten, horses tend to whirl and bolt when there is a perception of danger. Thus, utilization of the fNIRS equipment had the potential to create anxiety, including sudden escape movements among the horses, which could have endangered participants or staff. During the approximately 24 h of EAS provided in this study, the equines did not exhibit any fear-related behaviors.

Another important safety consideration was the human response to wearing the fNIRS equipment while participating in unfamiliar EAS activities. Given the interest in utilizing EAS for veterans with PTSD and trauma histories [16], this study was designed to assess the use of these methodologies for that population. Participating in an unfamiliar, and potentially anxiety-provoking, activity while wearing physiological measuring devices could potentially result in an exacerbation of symptoms. The study staff did not observe any evidence of discomfort among participants and the psychological instruments revealed pre-to-post-intervention decreases in anxiety and negative affect along with increases in positive affect. Thus, these preliminary results suggest that these methodologies can be safely utilized among the population studied. This is particularly notable given that all of the subjects had a military-related disability, and most had psychiatric and/or medical comorbidity in addition to a diagnosis of PTSD.

Cortical activation, as assessed by real-time fNIRS, revealed distinct differences during equine interactions (Figure 2: grooming and leading) compared to meditation and cognitive processing exercises. Higher activation over time was observed in the prefrontal cortex during horse–human interactions, whereas meditation and cognitive processing activities were associated with comparatively reduced and stable neural activation patterns, as noted in Figure 2. Increased prefrontal activity could be associated with various functions associated with cortical subregions, such as working memory, attention, emotional regulation, motivation, and movement planning [42]. In contrast, decreased activation may represent reduced cognitive demands, such as decreased attention, working memory, and executive function. While this study does not provide information about the impacts of EAS activities on cognitive function, by demonstrating the feasibility of using this modality to study EAS, it lays the groundwork for future fNIRS studies that can assess activation and functional connectivity of cortical subregions during EAS activities and thus further explore the neural mechanisms underlying the benefits associated with EAS interventions.

Our findings are different than those reported by Zhu and colleagues [21], who conducted the only other neuroimaging study of EAS that we are aware of. However, this is likely, at least in part, due to differences in the EAS intervention provided as well as the neuroimaging modalities utilized. While we used real-time fNIRS, their work involved resting-state fMRI pre- and post-intervention. Further, they found increases in the functional connectivity of the subcortical caudate nucleus that would not be revealed by fNIRS, which can only provide information about cortical activation. Lastly, our study did not assess functional connectivity. Nonetheless, our findings indicate that brain activation changes occur during EAS and vary with the activity, as noted in Figure 2. Given the connections between the frontal cortex and the caudate by way of the cortico-basal ganglia circuitry [43], it is possible that changes in frontal cortex activation contribute to the changes in caudate functional connectivity. Future research combining real-time fNIRS and resting-state fMRI methodologies may be a useful approach to understanding the neural mechanisms underlying the benefits of EAS. Additional studies will also be needed to fully understand the neural processes underlying how EAS interventions may lead to decreased symptoms of PTSD, but our work and that of Zhu et.al [21] suggest that both cortical and subcortical mechanisms may be involved.

The psychological instruments completed pre- and post-session revealed decreased anxiety and negative affect (negative feelings), as well as increased positive affect (positive feelings) and psychological flexibility (aware and in the present moment, able to change). These results indicate that participation in the activity, and associated physiological monitoring, did not cause psychological distress for the participants. Further, these results are consistent with other studies of EAS that have demonstrated short-term improvements in anxiety [44,45,46], affect [30,44,47,48], and psychological flexibility [30,44]. While the analyses revealed a statistically significant change in all variables pre- and post-intervention, there was no carryover effect from Visit 1 to Visit 2. Thus, the noted improvements in emotional health were not sustained from one intervention visit to the next. This may indicate that multiple sessions are necessary to obtain longer-term effects or that this type of intervention produces short-term effects. However, a previous study demonstrated sustained change over time after completing eight sessions of an intervention that included horses [49].

A study methodological issue, discovered during the analyses phase of the project, prevented evaluating correlations between fNIRS and psychological instrument results. Specifically, because the psychological assessments were completed each visit prior to and after engaging in all four activities and the fNIRS data were collected continuously during all four activities, comparison between the data sets was not possible. Thus, while we were unable to complete this evaluation, our results suggest that future studies should utilize both physiological and psychological metrics in a study design that facilitates such correlational analyses. Future comparison studies, with a larger number of participants and separate condition/activity protocols, are warranted to determine which type of activity (horse, therapist, horse + therapist) is most likely to improve a participant’s psychological health and wellbeing and to determine the relationship between those changes and neural activity.

Lastly, the EAS activity components utilized in this study were chosen, in part, because they represent activities that are common to various EAS interventions. Thus, our results suggest that fNIRS methodologies may be utilized to study a variety of EAS interventions. However, these components are also specific to the Whispers with Horses EAS intervention [30], which was developed for veterans with trauma histories. Results reported herein support previous findings of psychological benefits associated with participation in this intervention and indicate that future neuroimaging studies of this specific intervention are warranted.

### Limitations

Several limitations should be considered when interpreting the study findings. First, this study was not randomized and there was no control group. Thus, selection bias is a concern, and cause-and-effect relationships cannot be established. Further, the subjects were all male veterans with a diagnosis of PTSD and the normative values for the psychological measures were not specifically based on veterans with PTSD. It is unknown whether the results will generalize to other populations, including other veteran populations. Also, evaluating the effects of the horse-focused activities versus the therapist-led activities on the psychological measures was not possible with the sample size. Regarding safety observations during the sessions, a formal coding process was not utilized. Thus, minor patterns of human or equine behaviors could have been missed. Lastly, as noted above, correlational analyses to establish relationships between psychological and fNIRS data were not possible due to study design limitations. Further, the time of day for the intervention was not standardized across visits, which is a limitation of the fNIRS data, as factors such as diurnal variations in arousal, hormonal fluctuations, or fatigue could influence brain activation. Future research should aim to schedule sessions at consistent times or include the time of day as a covariate to better control for potential timing effects. Nonetheless, this study achieved the primary goal of establishing fNIRS methodology as a feasible and safe method to evaluate neural mechanisms associated with EAS participation.

## 5. Conclusions

This proof-of-concept study suggests that fNIRS methodology can be safely used to assess human brain activation during common EAS intervention activities. The fNIRS methodology used in this study provided real-time data on participants’ neural activity patterns as they engaged in both equine and non-equine EAS activities. The methodology was well-tolerated by both veterans and equines and there was no evidence of any safety risks for the participants, staff, or equines. These results lay the groundwork for future fNIRS studies to explore possible neural mechanisms associated with EAS interventions in general and the Whispers with Horses intervention specifically. As the only neuroimaging methodology capable of assessing real-time brain activation of humans during participation in EAS activities, fNIRS may play a major role in future studies of EAS mechanisms of action.

## Figures and Tables

**Table 1:** Experimental conditions of the research intervention (40 min).

Activity	Facilitator(s)	Details	Role of Equine	Duration
(A) Equine grooming	ES	Instruction by the ES on how to properly groom a horse and subsequent practice by participant.	Receives grooming	Ten minutes
(B) Equine groundwork	ES	Instruction by the ES on groundwork and subsequent practice by participant.	Participates in activities	Ten minutes
(C) Mindfulness meditation	MH (ES participating in meditation)	MH provides a brief description of mindfulness and then leads a breath & body focused mindfulness meditation	Near activity but not directly involved	Ten minutes
(D) Processing/discussion	MH (ES participating in discussion)	MH facilitates discussion about experimental activities	Near activity but not directly involved	Ten minutes

ES = equine specialist; MH = mental health professional.

**Table 2:** Demographic and diagnostic characteristics of participants (*n* = 15).

Characteristic	*n*/*M*	%/*SD*
**Age**	40.5	7.5
**Male gender**	15	100%
**Race**		
White	12	80%
Black	1	0.7%
Asian	2	1.3%
**Military branch**		
Army	9	60%
Navy	1	0.7%
Marine Corps	3	20%
Air Force	2	1.3%
**Combat deployments**		
Yes	13	87%
No	2	13%
**Military-related disability**	15	100%
**Psychiatric diagnoses**		
PTSD	15	100%
Depressive disorder	5	33%
Anxiety disorder	3	20%
**Pain disorder**	9	60%
**Number of medical diagnoses**	4.7	2.6

**Table 3:** Psychological assessment results.

	T1	T2			
Assessment	M (SD)	M (SD)	T (df)	*p*	95% CI
STAI-Day 1	14.13 (3.23)	8.67 (2.71)	−7.24 (14)	<0.001	[−7.086, −3.847]
STAI-Day 2	13.71 (3.68)	9.14 (2.98)	−0.8955 (13)	<0.001	[−5.674, −3.469]
AAQ-II Day 1	37.73 (8.11)	34.07 (8.32)	−3.214 (14)	0.003	[−6.114, −1.220]
AAQ-II Day 2	36.71 (10.37)	32.64 (8.82)	−3.337 (13)	0.003	[−6.707, −1.436]
PANAS Positive-Day 1	30.93 (6.90)	36.20 (6.38)	4.861 (13)	<0.001	[2.857, 7.428]
PANAS Positive Day 2	27.79 (6.37)	34.46 (6.46)	3.692 (12)	0.001	[2.775, 10.764]
PANAS Negative * Day 1	Mdn = 14	Mdn = 12	Z = −3.20	0.001	[−7.500, −1.000]
PANAS Negative * Day 2	Mdn = 14.5	Mdn = 12	Z = −3.06	0.002	[−7.000, −1.500]

* Wilcoxon signed-rank test was used because the data violated the Shapiro–Wilk test of normality. Mdn = median rank.

## Data Availability

Data are contained within this article.
